# Synthetic Drug Use and Limitations of Hospital Drug Screening: A Case of Gamma-Hydroxybutyrate Toxicity

**DOI:** 10.7759/cureus.88974

**Published:** 2025-07-29

**Authors:** Lily D Rundquist, Emma Newquist, Erick A Boldt, Daniel Pacciulli, Victoria Echevarria, Tianna L Nelson, Azjaah Rogers, Rebecca Cherner

**Affiliations:** 1 Dr. Kiran C. Patel College of Osteopathic Medicine (KPCOM), Nova Southeastern University, Fort Lauderdale, USA; 2 Family Medicine, Broward Health Medical Center, Fort Lauderdale, USA

**Keywords:** drug overdose, drug overdose management, gamma-hydroxybutyrate, natural and synthetic illicit drugs, urine drug screen limitations

## Abstract

Gamma-hydroxybutyrate (GHB) is a drug that acts as a central nervous system depressant and is commonly known to be used recreationally. We present the case of a 31-year-old male patient with a past medical history of hypertension (HTN), polysubstance abuse, and previous fentanyl overdose who was brought in an unconscious state to the emergency department (ED) after a suspected GHB overdose. Upon arrival at the ED, the patient was obtunded and therefore medically sedated and intubated for airway protection. His initial laboratory workup revealed a negative serum drug screen and positive urine drug screen for methamphetamines. Both urinalysis and respiratory panel were unremarkable. Several hours later, the patient regained consciousness, became agitated, and self-extubated. The patient’s presentation of altered mental status, respiratory depression, and subsequent agitation was attributed to the GHB overdose; however, due to limitations on standard hospital drug screens, it was not detected on the initial work-up. This case aims to demonstrate the increase in the prevalence of GHB use and the importance of considering it in the differential diagnosis of suspected overdose patients. In addition, this case highlights the need for broader toxicology reports in hospital settings to improve the timely diagnosis and management of GHB-related overdoses.

## Introduction

Gamma-hydroxybutyrate (GHB) is an endogenous short-chain hydroxylated carboxylic acid that is a precursor and metabolite of the neurotransmitter gamma-aminobutyric acid (GABA) and acts as a depressant on the central nervous system (CNS) [1]. In the United States, it is Food and Drug Administration (FDA) approved as a treatment for narcolepsy-associated cataplexy, but also widely known to be used recreationally [2]. It originally gained popularity in the 1990s as both a growth-promoting supplement and club drug, but in 2000, it became listed as a schedule one drug due to its use in drug-facilitated sexual assault [2].

Multiple reports indicate that GHB was most prevalent in the 2000s, with a peak in both emergency department (ED) visits and poison control center reports related to GHB toxicity during that time [3]. Although overall use declined after the early 2000s, a study done in 2020 showed an increase in use, particularly in nightclub and festival attendees, suggesting an ongoing upward trend [3]. Furthermore, a study done in 2021 found that the average age of people who use GHB, require treatment from GHB use, or experience GHB-mortality is 20s or early 30s, although a small proportion who overdose are in their 40s and 50s [3].

Users of this drug have compared the effects to alcohol and ecstasy, mentioning its euphoric properties while noting that it does not lead to a hangover [3]. Though with its steep dose-response curve, users are more predisposed to overdose and experience unconsciousness or a coma that may last up to eight hours [3]. Of note, the concurrent use of amphetamines with GHB has been reported to increase the risk of seizure and is co-ingested by the user since it’s thought to decrease the recovery time of a GHB-related coma [4]. This case study aims to bring awareness to the limited availability of synthetic drugs such as GHB in standard drug panels, and why it is important to be vigilant of the increasing use of GHB and of how toxicity may present.

## Case presentation

A 31-year-old man with a past medical history of hypertension (HTN), polysubstance abuse, and previous fentanyl overdose was brought to the ED by emergency medical services (EMS) after being found unconscious outside his home by a neighbor. He was found with “crystals” and GHB. The patient completed a rehabilitation program after his last overdose in 2021 and was thought to be sober currently; however, he recently went through a divorce in December of 2024. His father disclosed that his son had been drinking alcohol more frequently in the last few months, but they had not been aware of any other substances.

He has no past surgical history or hospitalizations other than during his fentanyl overdose. He takes valsartan at home for his HTN and is allergic to penicillin. He has no family history. He is self-employed, working in shade installation, and has a history of fentanyl, GHB, marijuana, and alcohol use.

EMS placed a non-rebreather and nasal trumpet for airway protection and gave him six mg of naloxone, which did not improve his mental status. Upon arrival at the ED, he was sedated, intubated, and given two mg more of naloxone with no effect. He was hypertensive and bradycardic with a normal oxygen saturation on the non-rebreather. Physical examination was significant for an obtunded male with conjunctival injection, bilateral miosis, and mechanical breath sounds secondary to ventilator use. His cardiac exam showed a bradycardic rate and regular rhythm. His abdomen was soft, non-distended, and non-tender, with bowel sounds present. Pedal pulses were appreciated bilaterally. There was no extremity edema or cyanosis.

**Figure 1:**
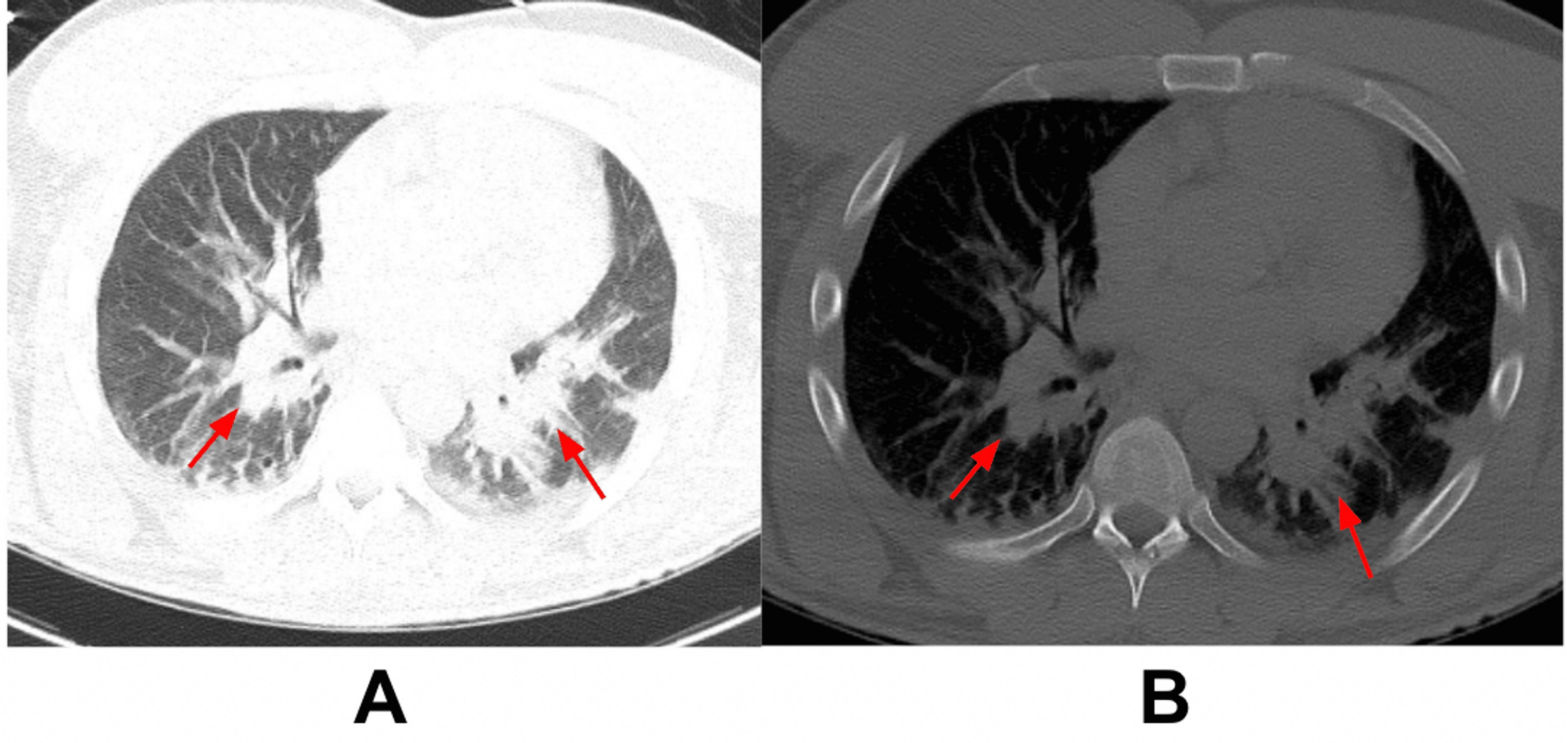
A and B show a CT chest without contrast depicting bilateral pneumonia, indicated by the red arrows.

Laboratory work-up included arterial blood gas (ABG) and venous blood gas (VBG), which showed respiratory acidosis with a pH of 7.31 and 7.26, respectively, and a carbon dioxide (CO2) of 49 and 68, respectively. Lactic acid, complete blood count (CBC), troponin, brain natriuretic peptide (BNP), partial thromboplastin time (PTT), and prothrombin time (PT) were all within normal limits (WNL). The serum drug screen was negative, but the urine drug screen was positive for methamphetamines. Urinalysis and respiratory panel were benign. Electrocardiogram (EKG) showed sinus bradycardia with possible left ventricular hypertrophy. CT brain and CT head/neck showed no acute process. The CT chest showed possible bilateral pneumonia (Figure 1). Blood and sputum cultures were collected.

**Figure 2:**
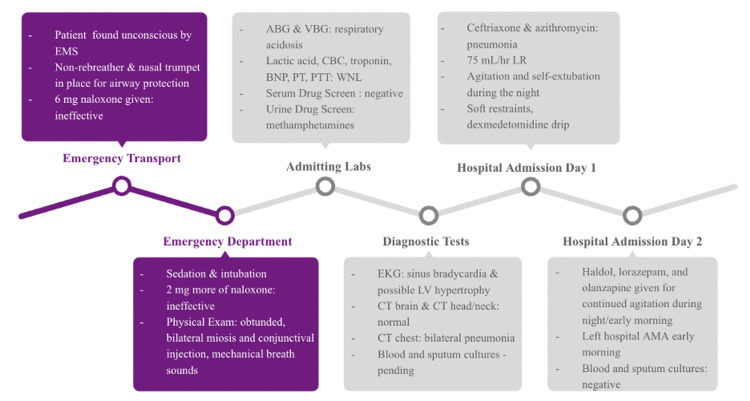
Summary of patient care from EMS arrival to discharge. EMS: Emergency medical services; LV: left ventricular; AMA: against medical advice; BNP: brain natriuretic peptide; PTT: partial thromboplastin time; PT: prothrombin time; WNL: within normal limits.

He was initially given ceftriaxone and azithromycin for the suspected pneumonia and started on a 75 mL/hr lactated ringer (LR). Around seven to eight hours later, the patient became conscious, highly agitated, and self-extubated. Soft restraints were placed on his upper extremities, and he was put on a dexmedetomidine drip. Due to continued agitation, he was given Haldol, lorazepam, and olanzapine at various times throughout the night. The patient left against medical advice (AMA) on hospital day two. Subsequent follow-up of the blood and sputum cultures was negative for any pathological bacterial growth. Figure 2 shows a summary of the patient’s care.

## Discussion

This case highlights the increasing prevalence of GHB overdoses and other synthetic drug use in the hospital setting. Despite the patient’s history of polysubstance abuse and a previous fentanyl overdose, his altered mental status raised a diagnostic challenge due to the limited availability of synthetic drug testing in standard hospital toxicology screens. The negative serum drug screen and positive urine drug test for methamphetamines demonstrate the limitations of standard hospital drug screens and their inability to detect newer synthetic drugs, ultimately emphasizing the need for expanded testing capabilities in emergency settings.

GHB has re-gained popularity due to its affordability, ease of ingestion, and rapid onset of euphoric and calming effects at lower doses [5]. The majority of users even perceive the drug as relatively harmless and comparable to alcohol [5]. However, at higher doses, GHB can induce profound CNS depression and autonomic instability, leading to bradycardia, hypotension, convulsions, respiratory failure, and possibly coma [5,6]. 

The patient’s presentation of obtundation, respiratory acidosis, and bradycardia is consistent with GHB toxicity, particularly given the absence of opioid responsiveness to multiple doses of naloxone. One hallmark of GHB toxicity is the rapid onset of coma, followed by a spontaneous recovery within three to eight hours [5,7]. Management remains largely supportive, with airway protection, cardiovascular monitoring, and cautious use of sedation for agitation following awakening [8]. In this case, the patient’s extreme agitation upon extubation required benzodiazepines and antipsychotic drug use, which aligned with documented cases of GHB withdrawal and post-intoxication delirium.

Unlike opioid withdrawal, which typically presents with autonomic hyperactivity, GHB withdrawal can present more severely and is more neuropsychiatric in nature due to extreme agitation being a typical feature upon awakening [7]. Withdrawal symptoms can emerge only hours after the last dose, progressing from anxiety, tremors, and diaphoresis to severe agitation and delirium within 24 hours [6,7]. The withdrawal profile closely resembles that of benzodiazepine or alcohol withdrawal, with autonomic instability and seizures occurring in more severe cases [7]. Table 1 shows a comparison of the substances previously mentioned. The patient’s post-extubation agitation, necessitating pharmacologic sedation, suggests possible early withdrawal, further complicating acute management.

**Table 1:** Comparison of withdrawal features between commonly abused substances This table summarizes key clinical characteristics of withdrawal syndromes associated with opioids, methamphetamine, GHB, alcohol, and benzodiazepines. Parameters include onset and peak of withdrawal, mood and autonomic symptoms, neurologic manifestations, risk of seizures or psychosis, lethality, and response to naloxone [6-11]. HTN: Hypertension; GHB: Gamma-hydroxybutyrate

Category	Opioids	Methamphetamine	GHB	Alcohol	Benzodiazepine
Onset of Withdrawal	6-12 hours (short-acting), 24-48 hours (long-acting)	1-2 days after last use	1-6 hours after last dose	6-25 hours after last drink	2-3 days (short-acting), 5-10 days (long-acting)
Peak Symptoms	2-3 days	2-7 days	24-72 hours	24-72 hours	1-2 weeks
Mood Symptoms	Anxiety, irritability, dysphoria, restlessness	Depression, anxiety, irritability	Anxiety, agitation, paranoia, panic	Anxiety, irritability, agitation	Anxiety, panic, irritability, depression
Autonomic Symptoms	Sweating, tachycardia, HTN, tachypnea, lacrimation, rhinorrhea, yawning	Fatigue, increased appetite, low energy	Sweating, bradycardia, HTN or hypotension, bradypnea	Sweating, tachycardia, HTN	Sweating, tachycardia, HTN
Neurological Symptoms	Dilated pupils, muscle aches, chills, goosebumps	Psychomotor slowing, poor concentration, vivid dreams, insomnia or hypersomnia	Tremors, confusion, delirium, hallucinations, rapid onset coma followed by spontaneous recovery within 3-8 hours	Tremors, confusion, delirium, hallucinations	Tremors, dizziness, perceptual disturbances, cognitive impairment. insomnia, tinnitus
Seizures	Rare	Rare	Common	Common	Common
Psychosis	Rare	Common	Common	Common	Common
Lethality	No	No	Yes	Yes	Yes
Response to Naloxone	Yes	No	No	No	No

Hospital-based drug screening often relies on urine immunoassays that target a limited panel of substances, including amphetamines, cocaine, marijuana, opiates, and phencyclidine [12]. While some expanded panels include benzodiazepines and synthetic opioids, GHB and other novel psychoactive substances are not routinely tested due to the rapid nature of newly developing synthetic compounds [12]. Moreover, because GHB has a rapid metabolism, its detection window is only a few hours, causing many cases to go undiagnosed unless specific testing is immediately requested and available. The pharmacological features of GHB are depicted in Table 2. Overall, this case demonstrates the critical need for broader toxicology screening, particularly in patients presenting with unexplained altered mental status and respiratory depression.

**Table 2:** Pharmacologic and toxicologic profile of GHB This table summarizes key pharmacologic properties of GHB, including its mechanism of action, dose ranges, half-life, metabolism, elimination pathways, receptor-specific EC₅₀ values, and detection window. Values are based on peer-reviewed pharmacokinetic and toxicology studies in both animal models and human subjects [1,13-17]. GHB: Gamma-hydroxybutyrate

Parameter	Description
Mechanism of Action	GHB receptor agonist (low dose) GABA_B_ receptor agonist (higher dose)
Therapeutic Dose Range	0.3-3.2 g
Lethal Dose for half of the population (LD_50_) in laboratory rodents	Approximately 2 g/kg → extrapolated to about 14 g in a 70 kg human
Effective concentration for half of the population (EC_50_)	Approximately 5mM (not well defined)
Onset of Action	15-30 minutes
Duration of Effects	3-4 hours
Half-Life	30-50 minutes
Metabolism	hepatic
Detection Window on Standard Urine Screen	< 6 hours (special test required for toxicology panel)

## Conclusions

This case demonstrates the growing use of GHB and other synthetic drugs, the challenges of limited toxicology screening, and the complexities of withdrawal in polysubstance users. The recent resurgence in GHB use highlights the importance of including it in the evaluation of overdose cases. The patient’s presentation of obtundation, respiratory depression, and subsequent agitation shows the complexities of GHB intoxication and withdrawal, as well as the patient’s rapid coma onset and quick awakening, a hallmark of GHB toxicity. Given its short-acting effects, clinicians must remain vigilant for GHB toxicity in unexplained altered mental status. With synthetic drug use increasing, expanding toxicology screening and clinician awareness are crucial. Standard drug panels miss GHB and other synthetic drugs, leading to underdiagnosis. Future efforts should focus on rapid testing and improved substance abuse education to enhance emergency care and patient outcomes.
